# Association of preexisting psychiatric disorders with post-COVID-19 prevalence: a cross-sectional study

**DOI:** 10.1038/s41598-023-27405-w

**Published:** 2023-01-07

**Authors:** Mayumi Kataoka, Megumi Hazumi, Kentaro Usuda, Emi Okazaki, Daisuke Nishi

**Affiliations:** 1 Department of Public Mental Health Research, National Institute of Mental Health, National Center of Neurology and Psychiatry, 4-1-1 Ogawahigashicho, Kodaira, Tokyo 187-8553 Japan; 2 Department of Sleep-Wake Disorder, National Institute of Mental Health, National Center of Neurology and Psychiatry, 4-1-1 Ogawahigashicho, Kodaira, Tokyo 187-8553 Japan; 3 Department of Mental Health, Graduate School of Medicine, The University of Tokyo, 7-3-1 Hongo, Bunkyo-ku, Tokyo, 113-0033 Japan

**Keywords:** Psychology, Medical research

## Abstract

Evidence demonstrating the association of preexisting psychiatric disorders with post-COVID-19 is limited. We aim to investigate the association using larger sample sizes and more extended postinfection periods than previous studies. A total of 6015 (response rate = 77.5%) COVID-19 survivors were surveyed using a self-administered questionnaire from July to September 2021. Poisson regression analysis with robust error variance was performed to estimate post-COVID-19 prevalence ratios (PRs) with or without preexisting psychiatric disorders. Participants with preexisting psychiatric disorders numbered 1067 (17.7%), and with post-COVID-19 were 2149 (35.7%). Post-COVID-19 PR with preexisting psychiatric disorders was 1.09 (95% CI 1.02–1.18, p = 0.013). The interaction between preexisting psychiatric disorders and postinfection periods was significant (p for interaction < 0.001). The subgroup analysis showed that those with preexisting psychiatric disorders might be at greater prolonged risk of post-COVID-19 than those without the disorders. These findings suggested that preexisting psychiatric disorders were associated with an increased post-COVID-19 risk, and post-COVID-19 with preexisting psychiatric disorders might prolong even if time passes.

## Introduction

The new coronavirus disease (COVID-19) has infected many people worldwide, with a still-growing number of people getting infected. As of May 22, 2022, 520 million people worldwide have been infected, and 6.3 million people have died^1^. Many people infected with the virus have been reported to exhibit persistent symptoms after COVID-19 and are called post-COVID-19. The World Health Organization (WHO) defined these symptoms as “usually 3 months from the onset of COVID-19 with symptoms that last for at least 2 months and cannot be explained by an alternative diagnosis. Common symptoms include fatigue, shortness of breath, and cognitive dysfunction but also others that generally have an impact on everyday functioning. Symptoms may be new onset, following initial recovery from an acute COVID-19 episode, or persist from the initial illness. Symptoms may also fluctuate or relapse over time”^2^. A previous systematic review showed that 72.5% of patients had persistent symptoms after 2 months of diagnosis or onset of or admission for COVID-19 or after 1 month of discharge or recovery^3^. Another previous systematic review revealed that 54% of patients had persistent symptoms of COVID-19 longer than 6 months after infection or discharge from the hospital^4^. Symptoms reduced the quality of life (QOL) of COVID-19 survivors^5–7^. Systematic review and meta-analysis previously showed that QOL was associated with mortality risk^8^. Thus, post-COVID-19 has been considered a public health problem and should be solved.

Preexisting psychiatric disorders and the postinfection period are considered important post-COVID-19 risk factors. A previous cross-sectional study of COVID-19 survivors showed that a chronic psychiatric condition was associated with not returning to the patient's usual health^9^. Another previous cross-sectional study revealed that a preexisting diagnosis of depression or anxiety was over-represented in those with fatigue post-COVID-19^10^. People with psychiatric disorders were associated with increased risks of chronic physical symptoms compared with those without psychiatric disorders based on previous cross-sectional studies^11–13^. Therefore, post-COVID-19 may be more prolonged in those with preexisting psychiatric disorders than in those without the disorders.

However, previous studies that investigated the association of preexisting psychiatric disorders with post-COVID-19 had small sample sizes and short postinfection periods: the median interval from test to interview date was 16 days (the interquartile range was 14–19 days), and the median interval of symptom assessment was 10 weeks after the initial COVID-19 symptoms^9,10^. Therefore, the evidence about the association of preexisting psychiatric disorders with post-COVID-19 and the difference in the risk of a more extended postinfection period with or without preexisting psychiatric disorders are still limited. Moreover, a previous study showed that the rate of medication use among people with psychiatric symptoms was low in Japan^14^. The clinical data of medical settings would not be sufficient to determine the prevalence and the course of post-COVID-19. Therefore, confirming the association between preexisting psychiatric disorders and post-COVID-19 among people who would not have used medical care after recovering from COVID-19 infection is necessary. Our previous study showed that the courses of depressive and anxiety symptoms after the infection of COVID-19 were more severe in COVID-19 survivors with preexisting psychiatric disorders than in those without the disorders^15^. However, the association between preexisting psychiatric disorders and physical symptoms of post-COVID-19 is still unknown.

This study aims to investigate the association between preexisting psychiatric disorders and physical symptoms of post-COVID-19 and the interaction of preexisting psychiatric disorders and postinfection period on physical symptoms of post-COVID-19 in larger sample size and more extended postinfection period than those of previous studies by an internet survey.

## Methods

### Participants

**Figure 1:**
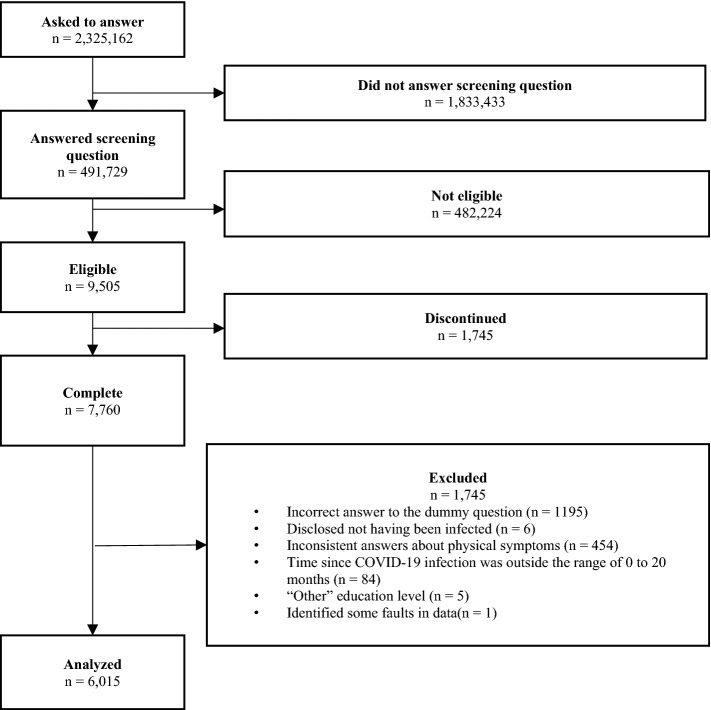
Participant flow chart.

Participants who have contracted the new coronavirus were surveyed using a web-based cross-section analysis from July to September 2021. The study sample was from the pooled panels of an internet research agency (Rakuten Insight, Inc.), which had approximately 2.2 million panelists in 2019. All participants provided web-based informed consent at registration. Only the participants who responded “yes” to the first question, “Have you ever been infected with COVID-19?” were asked to complete the questionnaire. We excluded participants who completed the questionnaire (n = 7760) who (1) incorrectly answered the dummy question (n = 1195); (2) disclosed not having been infected during the survey (n = 6); (3) gave inconsistent answers about physical symptoms (n = 454); (4) gave improbable answers about the postinfection period (not within 0–20 months) judging from the date of confirmed the first case of COVID-19 infection in Japan (January 15, 2020)^16^ (n = 84); (5) answered in the open-ended section, which could not categorize existing choices of the questionnaire (n = 5); and (6) identified some fault in data (n = 1). The participant flow chart is shown in Fig. 1. Finally, 6015 individuals (response rate = 77.5%) were included in the analyses. The study was in accordance with the Declaration of Helsinki. All methods were performed in accordance with the relevant guidelines and regulations. The participation was anonymous. A credit point that could be used for internet shopping and cash conversion was provided for participants with an incentive. We used the term gender according to the SAGER guidelines^17^. This study was approved by the Ethical Board of the National Center of Neurology and Psychiatry in Japan (A2021-34).

### Measurement

#### Outcome variables

##### Post-COVID-19

Post-COVID-19 was dichotomized into “yes” and “no.” We identified post-COVID-19 based on the WHO definition^2^. We asked the question: “What symptoms do you have?” Participants could choose multiple answers from the options of symptoms based on the WHO definition of post-COVID-19. Responses were categorized as “yes” when participants chose any one of these considered physical symptoms (menstrual and period problems, altered smell, altered taste, blurred vision, chest pain, cough, dizziness, fatigue, [intermittent] fever, gastrointestinal issues [diarrhea, constipation, or acid reflux], headache, muscle pain or spasms or neuralgias, shortness of breath, tachycardia or palpitations, and tinnitus and other hearing issues). Post-COVID-19 that participants described in the open-ended section were reviewed by four researchers and allocated the type of symptoms they should be applied to.

#### Exposure variables

##### Preexisting psychiatric disorders

Preexisting psychiatric disorders were categorized by the answers to the question: “Have you ever been diagnosed with or experienced psychiatric problems before the COVID-19 pandemic?” Participants chose multiple answers from the following options of symptoms: “Nothing,” “Depressive disorder,” “Bipolar disorder,” “Panic attack or panic disorder,” “Anxiety disorder or anxiety-related problems (e.g., hypersensitivity, worry, fear, obsessive–compulsive symptoms),” “Alcohol use disorder or alcohol abuse/dependence,” “The use of illicit substances or psychotropics without prescription,” “Burnout syndrome,” and “Others” (with an optional comment field). Preexisting psychiatric disorders were dichotomized into “yes” and “no” based on the Diagnostic and Statistical Manual of Mental Disorders Fifth Edition (DSM-5)^18^. Those who chose any one of these disorders (depressive disorder, bipolar disorder, panic attack or panic disorder, anxiety disorder, or anxiety-related problems [e.g., hypersensitivity, worry, fear, and obsessive–compulsive symptoms]) were categorized into the group “yes” preexisting psychiatric disorders. Those who chose options only “Nothing,” “The use of illicit substances or psychotropics without prescription,” or “Burnout syndrome” were categorized into the group “no” preexisting psychiatric disorders. We considered that “The use of illicit substances or psychotropics without prescription” did not meet the diagnostic criteria for alcohol use disorder without the information on duration, amount of use, and symptoms. Moreover, the DSM-5 does not include Burnout syndrome. Among those who chose only “Others,” those whose comments in the comment field matched the DSM-5 diagnosis were categorized into the group “yes” preexisting psychiatric disorders.

#### Covariates

Covariates were selected based on the basis of previous studies of associated factors of post-COVID19^18–22^ and psychiatric disorders.

##### Kessler6 (K6)

Psychological distress was measured using K6^23^. It consists of six items assessing the frequency of psychological distress occurring in the last 30 days. The response choices are from 0 (none of the time) to 4 (all of the time), and the total score ranges from 0 to 24. The total scores of K6 were categorized as follows: no (≤ 4), slight or moderate (5–12), and severe (≥ 13).

##### Generalized Anxiety Disorder-7 (GAD-7)

Anxiety symptom was measured using GAD-7^24^. It consists of seven items assessing the frequency of symptoms of anxiety occurring in the last 2 weeks. The response choices are from 0 (not at all) to 3 (nearly every day), and the total score ranges from 0 to 21. The GAD-7 total scores were categorized as follows: no (≤ 4), slight (5–9), moderate (10–14), and severe (≥ 15).

##### Other covariates

We measured other covariates as follows: postinfection period (< 1 month, ≥ 1 to < 3 months, ≥ 3 to < 6 months, ≥ 6 to < 12 months, or ≥ 12 months), early dyspnea of COVID-19 (yes or no), more than five early symptoms of COVID-19 (yes or no)^21^, treatment of COVID-19 (no hospitalization, hospitalization without the ICU stay, or hospitalization with the ICU stay), age (20–29, 30–39, 40–49, 50–59, or ≥ 60 years), gender (male, female, or other), educational attainment (high school or lower, some college [e.g., junior college], or university graduate or higher), work status (self-employed, permanent employment, temporary employment, unemployed, or student), cohabitation (yes or no), and medical history (yes or no). The responses were categorized as “yes” when participants answered that they had any of the following medical histories during the survey: hypertension, diabetes, asthma, bronchitis or pneumonia, atopic dermatitis, angina pectoris, cardiac infarction, chronic obstructive pulmonary disease, or cancer.

### Statistical analyses

The post-COVID-19 prevalence ratios (PRs) were estimated using Poisson regression analysis with a robust error variance. We used this model because the post-COVID-19 prevalence is over 10%, and the odds ratio could overestimate the PRs^25^. Variance inflation factor (VIF) was used to check for multicollinearity. Most of the VIF values were less than 2, and the mean VIF of the model was < 2. We entered K6 and work status as covariates because we considered that these were important covariates, although they were over 2 (K6: ≥ 13 = 2.18; work status: self-employed = 2.28 and permanent employment = 2.01). We also estimated the interaction between preexisting psychiatric disorders and postinfection periods. We then conducted a subgroup analysis by postinfection periods because the interaction between preexisting psychiatric disorders and postinfection periods was significant. All analyses were performed using Stata 17.0 (Stata Corp, College Station, TX, USA).

## Results

### Characteristics of the participants

Table 1 shows the participants' characteristics (n = 6015). Participants with preexisting psychiatric disorders were 1067 (17.7%), and participants with any post-COVID-19 were 2149 (35.7%). There was a significant difference in post-COVID-19 prevalence according with or without preexisting psychiatric disorders (52.7% vs. 32.1%, p < 0.001). The number of participants in each postinfection period was the following: (< 1 month = 1138 (18.9%), ≥ 1 to < 3 months = 1443 (24.0%), ≥ 3 to < 6 months = 1369 (22.8%), ≥ 6 to < 12 months = 1563 (26.0%), ≥ 12 months = 502 (8.3%), respectively. Most of the ages of participants were 30–39 (1504, 25.0%) and 40–49 (1581, 26.3%). Of the total number of participants, 3441 (57.2%) were male, 2554 (42.5%) were female, and 20 (0.3%) were other.

**Table 1:** Baseline characteristics of study participants by existence of post-COVID-19 (n = 6015).

	Total	%	Post-COVID-19: NO (n = 3866)		Post-COVID-19: YES (n = 2149)		χ^2^ p-value
			n	%	n	%	
**Preexisting psychiatric disorders**							
No	4948	82.3	3361	67.9	1587	32.1	< 0.001
Yes	1067	17.7	505	47.3	562	52.7	
**Postinfection period**							
< 1 month	1138	18.9	499	43.9	639	56.1	< 0.001
≥ 1 to < 3 months	1443	24.0	879	60.9	564	39.1	
≥ 3 to < 6 months	1369	22.8	993	72.5	376	27.5	
≥ 6 to < 12 months	1563	26.0	1138	72.8	425	27.2	
≥ 12 month	502	8.3	357	71.1	145	28.9	
**Early dyspnea of COVID-19**							
No	4890	81.3	3422	70.0	1468	30.0	< 0.001
Yes	1125	18.7	444	39.5	681	60.5	
**More than five early symptoms of COVID-19**							
No	3398	56.5	2590	76.2	808	23.8	< 0.001
Yes	2617	43.5	1276	48.8	1341	51.2	
**Treatment of COVID-19**							
No hospitalization	4545	75.6	3107	68.4	1438	31.6	< 0.001
Hospitalization (without ICU stay)	1339	22.3	710	53.0	629	47.0	
Hospitalization (with ICU stay)	131	2.1	49	37.4	82	62.6	
**K6**							
≤ 4	3636	60.4	2785	76.6	851	23.4	< 0.001
5–12	1831	30.4	872	47.6	959	52.4	
≥ 13	548	9.1	209	38.1	339	61.9	
**GAD-7**							
≤ 4	4292	71.4	3188	74.3	1104	25.7	< 0.001
5–9	1033	17.2	450	43.6	583	56.4	
10–14	457	7.6	153	33.5	304	66.5	
≥ 15	233	3.9	75	32.2	158	67.8	
**Age years**							
20–29	1192	19.8	816	68.5	376	31.5	< 0.001
30–39	1504	25.0	951	63.2	553	36.8	
40–49	1581	26.3	956	60.5	625	39.5	
50–59	1189	19.8	735	61.8	454	38.2	
≥ 60	549	9.1	408	74.3	141	25.7	
**Gender**							
Male	3441	57.2	2276	66.1	1165	33.9	0.001
Female	2554	42.5	1581	61.9	973	38.1	
Other	20	0.3	9	45.0	11	55.0	
**Education**							
High school or lower	1355	22.5	835	61.6	520	38.4	0.009
Some college	1295	21.5	813	62.8	482	37.2	
University graduate or higher	3365	55.9	2218	65.9	1147	34.1	
**Work status**							
Self-employed	861	14.3	555	64.5	306	35.5	0.001
Permanent employment	3183	52.9	2087	65.6	1096	34.4	
Temporary employment	1052	17.5	616	58.6	436	41.4	
Unemployed	870	14.5	577	66.3	293	33.7	
Student	49	0.8	31	63.3	18	36.7	
**Cohabitation**							
No	1165	19.4	799	68.6	366	31.4	0.001
Yes	4850	80.6	3067	63.2	1783	36.8	
**Medical history**							
No	4418	73.4	2921	66.1	1497	33.9	< 0.001
Yes	1597	26.6	945	59.2	652	40.8	

*K6* Kessler6, *GAD-7* Generalized Anxiety Disorder-7.

### PR of post-COVID-19 with preexisting psychiatric disorders

The adjusted PR of post-COVID-19 with preexisting psychiatric disorders was 1.09 (95% CI 1.02–1.18, p = 0.013) (Table 2).

**Table 2:** The prevalence ratios (PRs) of post COVID-19 symptoms estimated using Poisson regression analysis with robust error variance (n = 6015).

	Crude				Adjusted				Adjusted^c^			
	PR	95% CI		p-value	PR	95% CI		p-value	PR	95% CI		p-value
**Preexisting psychiatric disorders**												
No	Ref				Ref				Ref			
Yes	1.64	1.53	1.76	< 0.001	1.09	1.02	1.18	0.013	0.88	0.79	0.97	0.015
**Postinfection period**												
< 1 month	Ref				Ref				Ref			
≥ 1 to < 3 months	0.70	0.64	0.76	< 0.001	0.70	0.65	0.76	< 0.001	0.68	0.63	0.74	< 0.001
≥ 3 to < 6 months	0.49	0.44	0.54	< 0.001	0.51	0.47	0.56	< 0.001	0.48	0.44	0.53	< 0.001
≥ 6 to < 12 months	0.48	0.44	0.53	< 0.001	0.53	0.48	0.57	< 0.001	0.47	0.42	0.52	< 0.001
≥ 12 month	0.51	0.44	0.60	< 0.001	0.55	0.48	0.63	< 0.001	0.47	0.40	0.55	< 0.001
**Preexisting psychiatric disorders × postinfection period**									1.15	1.09	1.21	< 0.001

^a^PRs were estimated using Poisson regression analysis with robust error variance.

^b^Adjusted: early dyspnea of COVID-19, more than five early symptoms of COVID-19, treatment of COVID-19, K6, GAD-7, age, gender, education, work status, cohabitation, and medical history.

^c^Interaction: preexisting psychiatric disorders × postinfection period included in the statistical model.

### Interaction between preexisting psychiatric disorders and postinfection periods

**Figure 2:**
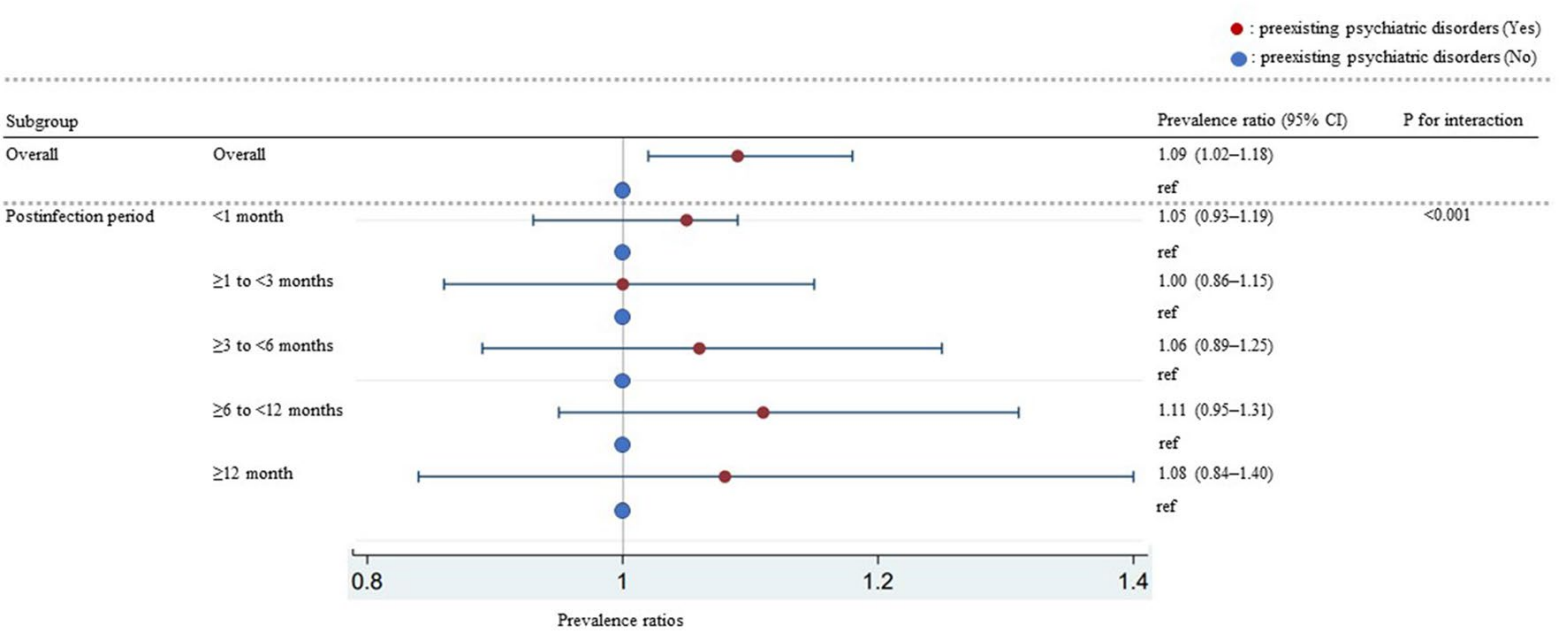
Result of subgroup analyses by postinfection periods (n = 6015).

The interaction between preexisting psychiatric disorders and postinfection periods was significant (p for interaction < 0.001) (Table 2). The subgroup analysis by postinfection period showed that PRs of with preexisting psychiatric disorders for reference (without preexisting psychiatric disorders) in each postinfection period were following (< 1 month: 1.05, 95% CI 0.93–1.19; ≥ 1 to < 3 months: 1.00, 95% CI 0.86–1.15; ≥ 3 to < 6 months: 1.06, 95% CI 0.89–1.25; ≥ 6 to < 12 months: 1.11, 95% CI 0.95–1.31; ≥ 12 months: 1.08, 95% CI 0.84–1.40) (Fig. 2).

## Discussion

This study investigated the association between preexisting psychiatric disorders and post-COVID-19 and the interaction of preexisting psychiatric disorders and postinfection periods using a web-based cross-sectional survey. The results showed that preexisting psychiatric disorders were associated with a slight increase in PR of post-COVID-19. Preexisting psychiatric disorders might tend to be at greater prolonged risk of post-COVID-19 than those without preexisting psychiatric disorders, except for ≥ 1 to < 3 months.

The result that preexisting psychiatric disorders were associated with an increase in PR of post-COVID-19 was consistent with previous cross-sectional studies about COVID-19 survivors that showed preexisting psychiatric disorders were associated with not returning to the patient's usual health or over-represented fatigue as post-COVID-19^9,10^. Previous studies suggested that current psychiatric disorders would cause physical symptoms^26,27^. However, the association of preexisting psychiatric disorders with post-COVID-19 was significant after adjustment for current depressive and anxiety symptoms in this study. It might be better to pay attention to the difference in the clinical course of post COVID-19 with or without preexisting psychiatric disorders. Previous meta-analyses showed that levels of inflammatory cytokines increased in patients with psychiatric disorders compared with controls^28,29^. Moreover, a previous study showed that the levels of inflammatory cytokines also increased in patients with post-COVID-19^30^. The overproduction of inflammatory cytokines could play an essential role in the cause and progress of various diseases^31^. The activation of the inflammatory response system caused by psychiatric disorders might be associated with higher PR among the participants with preexisting psychiatric disorders.

Preexisting psychiatric disorders might be at greater prolonged risk of post-COVID-19 than those without preexisting psychiatric disorders. The result could be consistent with previous studies that people with psychiatric disorders were associated with increased risks of chronic physical symptoms compared with those without psychiatric disorders^12,13^. There are some possible factors to prolong physical symptoms among people with psychiatric disorders. Previous studies, for example, suggested that current psychiatric symptoms^32^, the psychological factor of fear–avoidance^33,34^, and the neuropsychological factor of pain sensitivity^35^ were associated with chronic physical symptoms. However, these associations are still unclear. Further study will be needed to investigate the mechanism of preexisting psychiatric disorders, post-COVID-19, and both biological and nonbiological factors.

Unexpectedly, only in the postinfection period was ≥ 1 to < 3 months, the trend of greater risk of post-COVID-19 with preexisting psychiatric disorders was not observed. A previous study suggested that the persistence of physical symptoms differed with the type of psychiatric disorder^36^. The course of physical symptoms of post-COVID-19 might differ depending on the type of preexisting psychiatric disorders. Further research is needed to determine the relationship between the type of preexisting psychiatric disorders and the severity and course of physical symptoms of post-COVID-19 to identify populations needing recovery support.

## Limitations

This study had several limitations: first, this study was conducted using an internet survey. Participants were extracted from individuals who were registered as monitors of the research agency and agreed to call for research cooperation. Participants who were concerned with post-COVID-19, psychological distress, or psychiatric conditions would be more likely to respond to the survey, which could result in an overestimation of post-COVID-19 and psychiatric conditions. Therefore, the generalizability may be limited because of the possibility of sampling bias. Second, this study used self-administered questionnaires. There is a consistency limitation with the clinical definition of preexisting disorders and post-COVID-19. The limitation could also result in an overestimation of the prevalence of preexisting disorders and post-COVID-19 because of the same reason for sampling bias. Moreover, recall bias could occur if participants with post-COVID-19 had cognitive decline, which may be more common in those with preexisting psychiatric disorders. The limitation could also affect the results. Third, this study used a dichotomous variable about the presence of post-COVID-19. A previous cross-sectional study pointed out that the strength of associated psychiatric symptoms with physical symptoms differed in each type of physical symptom^37^. The association between preexisting psychiatric disorders and post-COVID-19 may differ in each type of symptom. Fourth, this study did not evaluate the difference in the type of new coronavirus variant in each participant. Patients infected with the Omicron variant would not be included because the end date of this survey was September 2021. The post-COVID-19 impact may vary depending on the new coronavirus variant infection type. Fifth, there would be unmeasured confounding because the mechanisms of post-COVID-19 are still unknown. It might affect the result of this study. Sixth, it is impossible to determine the causal relationship between preexisting psychiatric disorders and post-COVID-19 because this was a cross-sectional study. Moreover, we did not perform a pretest of the prevalence of physical symptoms before COVID-19 infection. The association of physical symptoms and post-COVID-19 due to preexisting psychiatric disorders may reflect the previous physical symptoms related to preexisting psychiatric disorders, not the physical symptoms of post-COVID-19.

## Conclusions

Our analyses suggested that preexisting psychiatric disorders of COVID-19 survivors were associated with an increased the risk of physical symptoms of post-COVID-19. Moreover, preexisting psychiatric disorders might be at greater prolonged risk of post-COVID-19 than those without preexisting psychiatric disorders.

## Data Availability

The data that support the findings of this study are available from the corresponding author upon reasonable request.
